# Flipped classroom with teams-based learning in emergency higher education: methodology and results

**DOI:** 10.1007/s10639-022-11339-3

**Published:** 2022-11-04

**Authors:** Konstantinos Antonis, Petros Lampsas, Ioannis Katsenos, Spyros Papadakis, Stella-Maria Stamouli

**Affiliations:** 1 Department of Physics (Distributed Systems and Networks Research Lab), University of Thessaly, Lamia, Greece; 2 Department of Digital Systems, University of Thessaly, Lamia, Greece; 3 Department of Management Science and Technology, School of Economics and Business, University of Patras, Lamia, Greece; 4 School of Science & Technology, Informatics Program of Studies, Hellenic Open University, Patras, Greece; 5 Computer Engineering Program of Studies, University of Thessaly, Lamia, Greece

**Keywords:** Blended learning, Flipped Classroom, Teams-based Learning, Formative Assessment

## Abstract

During the pandemic period, most of the universities shifted their curricula into fully distance learning models. Due to these Emergency Remote Education circumstances, we adopted the application of Flipped Classroom model combined with Team-based Learning pedagogical strategy in four Computer Engineering courses. Our approach was reinforced by employing LAMS Learning Activities Management System in conjunction with interactive video services. Results of the application of this approach reveal enhanced student engagement with learning resources and improved achievement when compared to the traditional, in-class, conduction of the same courses. Moreover, students report positive estimation of the adopted approach.

## Introduction

During the academic year 2020–2021, Greek Universities conducted most of their courses online due to COVID-19 pandemic. The Emergency Remote Education was performed using synchronous and asynchronous distance learning activities. Asynchronous distance learning tools were used mainly as repositories of educational material, while synchronous learning tools were used to replace teacher-centred instruction and offer opportunities for collaboration.

However, Distance Education is not simply a medium switch: a mere transfer of the education process that takes place in the amphitheater or in a laboratory to a synchronous distance learning platform does not suffice. Distance education has its own methodology, specific characteristics and requirements that must be satisfied to be effective and efficient. One of the key components in a distance learning setting is the interaction between the students and the educational material, which nowadays can be easily achieved through advanced learning technologies (Gunawardena & McIsaac, 2004).

According to the Flipped Classroom (FC) model (Bergmann & Sams, 2014), students undertake a more active role in the learning process. Teachers are mainly facilitators of the learning process and are available for students’ requests. The students should prepare at home, before the class, watching mainly interactive videos and teacher-selected learning material. During the face to face in classroom or synchronous online class time, students, either individually or in small groups, solve problems, elaborate on difficult concepts, and participate in learning activities scheduled by the teacher (Hamdan et al., 2013; Tucker, 2012; Papadakis et al., 2019). The teacher adapts the lesson and the virtual room equipment according to the needs and learning styles of his students and plans individual and group learning activities. The teacher also encourages, supports, and supervises the learning activities and undertakes an advisory and inspirational role. As students work independently in an FC environment, the teacher’s role is particularly critical, due to the different needs of the students.

Team-based learning (TBL) is a pedagogical strategy where students work through problems in small groups. This process, when carefully designed and monitored, motivates, and engages students by holding them accountable to themselves and one another. According to Huggins et al. (2015), this strategy is flexible enough to be implemented in classes of varying sizes including large lecture courses.

In our approach we decided to explore and evaluate the advantages that collaboration brings to the FC model by combining it with TBL. We used LAMS Learning Activities Management System (Dalziel, 2003) in conjunction with interactive video services to apply our method which is described in detail in Table 1 in the Methodology section to maximize the students’ learning experience.

**Table 1:** Combination of Flipped Classroom and Team-based Learning

1. Students complete pre-class readings and/or other assignments.
2. At the beginning of the synchronous distance education, teachers briefly clarify topics and answer student questions.
3. Students complete an Individual Readiness Assurance Test (iRAT) as an indication of what they learned from their pre-class assignments.
4. After completing this assessment, students collaborate to complete the Team Readiness Assurance Test, tRAT, and, if applicable, apply knowledge to solve Computer Engineering problems.
5. Answers to Readiness Assurance tests are revealed and students are asked to put forward any questions emerged by this procedure.
6. Teacher answers questions and clarifies issues for which students’ answers reveal lack of understanding.

In the Emergency Remote Distance Education context, we chose to shift the education model towards blended learning and the FC approach combined with Teams-based Learning. The reasons for this choice were two: the first one was to strengthen the individualized distance education process with suitable distance learning techniques and collaborative learning. The second one was to perform an experiment in real conditions and on a large scale, investigating the learning outcomes that the combined approach can have in tertiary education context.

In the following sections we present a brief literature review and the methodology adopted to conduct the courses with blended approach, and we elaborate on the evaluation of the proposed methodology and the corresponding learning outcomes. We conclude with plans on future work that further expand and explore the combination of FC with TBL.

## Related work

In distance learning as well as in blended learning settings students need to develop competencies that will allow them to work autonomously (Moore, 1973). It is crucial for each student to undertake responsibility for his own learning path, develop initiatives and commit to activities that lead to learning. Furthermore, it is considered important to combine student autonomy with collaborative skills invigoration. Inevitably the adoption of the flipped classroom approach leads to a shift in the role of the teacher. In an FC environment, teachers create interactive videos, share educational resources, facilitate the learning process, thus becoming, to a great degree, a “guide to the student” (Baker, 2000). While students work on assignments, the teacher provides personalized and group support. Teachers who are familiar with the traditional instructional model face difficulties, while the same holds for students who find it difficult to function in a self-regulated learning environment (Kim et al., 2014). In the following paragraphs we present research findings on the effectiveness of the application of FC approaches mainly in tertiary education and we briefly elaborate on the derived results focusing on comparing student achievement in FC and traditional approaches.

In Giannakos et al. (2016), an overview of the advantages that result from the application of FC model can be found. Unlike teacher-centered instruction where the presentation of the material is done once, the FC model allows students to repeatedly review the interactive videos or to skip parts of the material that they have already mastered. Interactive videos are not meant to replace the teacher but, on the contrary, provide ample time for personal contact between teacher and student (Bergmann & Sams, 2014; Van Wart et al., 2020) investigates various concepts identified in the literature as critical success factors for online learning from the students’ perspective, and then determines their hierarchical significance.

Yang et al. (2019) applied rotational blended learning in Computer Engineering students, and they studied the results of this approach in fostering their research and analytical skills. They used two groups of students: one that attended the “Systems Engineering Technologies” course according to the rotational blended learning method and the previous year’s students that attended the course using traditional teaching methods (as a control group). For the rotational blended learning procedure three elements were used: e-learning, mini-projects and seminars. They evaluated their approach by applying classroom observation, student surveys, and results, and the results demonstrated that the rotational blended learning approach outperformed the traditional one. Xu et al., (2021) flipped the classroom by implementing an online student-centered approach as well as small private online courses. By using a multiple regression model authors argue that students’ performance can be inferred by their behavior in the blended learning part of their approach.

The work of Al-Samarraie et al. (2020) presented an overview of the best practical applications of FC in Universities and in different scientific fields. The results were encouraging for some scientific fields, while for others there was no substantial gain from using the FC model. The work of Amresh et al., (2013) and Velegol et al., (2015), showed that the overall performance and effectiveness of students improved, even in cases where students felt that the FC model was particularly heavy, in terms of workload for them. Another study by Birgili et al., (2021) was conducted to reveal the trends and outcomes in research into the FC approach published between 2012 and 2018. Most of the studies reviewed were conducted with students in the subject area of education and medicine. The outcomes showed an increase in students’ performance and positive influence on cognitive and affective domain as well as in soft skills.

DeLozier et al. (2017) presented a variety of approaches to the flipped classroom model and investigated learning activities often used in these settings. They argued that video instruction does not result in improved learning performance of itself but may save time for engaging students to in-class activities resulting in enhanced learning outcomes. Regarding other learning activities frequently found in FC settings (e.g., quizzes, clicker questions, etc.) the authors argued that they must be adapted properly to the learning environment to enhance learning. They also mention that other forms of active learning (e.g., collaborative and problem-based learning) can enhance the FC process.

Troya et al., (2021) deduced that FC can improve the learning outcomes in undergraduate Computer Science lab sessions. Their results showed that lab flip can make time for more practice and collaboration between students. As far as student motivation is concerned, significant improvement was observed when compared to face-to-face methods. Hussain et al., (2020) adopt a constructivist student-centred approach to flip the classroom in an engineering course. They used a fuzzy logic-based approach for the qualitative survey responses given by the students. Based on the analysis of their results, authors found statistically significant improvement in learning outcomes and student engagement.

The work of Wang & Zhu (2019) showed that students in an online course conducted through FC had better performance than their colleagues who attended it in the traditional way. The work of McCredden et al., (2017) showed that the FC model can be used to develop students’ problem-solving skills and to better understand engineering courses. The work of Zainuddin & Halili (2016) presents the cumulative results of 20 applications of FC in higher education. It mainly focuses on the methodology that was applied, on the tools that were used, while qualitative analysis of the students’ progress during the semester is presented, with encouraging results. In contrast, the work of Hotle & Garrow (2016) shows that there are no significant differences between the FC model and traditional teaching in terms of student performance.

Hew (2020) conducts a comparative study on learning outcomes of online flipped classrooms vs. conventional flipped classrooms. For the purposes of the study two conventional flipped classes in the Faculty of Education were transferred online. The quantitative analyses of students’ learning outcomes showed insignificant difference between students that participated in the online flipped classes and students participated in the conventional flipped learning classes. The qualitative analyses of results recommended seven good practices for videoconferencing-assisted online flipped classrooms.

Van Alten et al., (2019) carried out a comparative survey on over a hundred approaches on flipped and non-flipped classrooms in secondary and postsecondary education. Although they reported no effect on student satisfaction as far as the learning environment is concerned, they detected a minor positive effect on learning outcomes. Results revealed that students in flipped classrooms benefitted when quizzes were added as an assessment method and class time session remained almost the same compared to non-flipped classrooms. Dalziel, B. et all. (2019) referred that medical departments explored the combination of Problem-based Learning and Team-based Learning approaches to better teach pre-clinical students the core concepts needed to proceed to a clinical environment.

Based on the findings of the aforementioned works, we conclude that the transformation of traditional courses to FC ones, results, in most of the studies, in enhanced learning outcomes. Obviously, application and evaluation of FC approaches attracts research attention, however, to the best of our knowledge, very few of the research works attempt to review the combination of FC with collaborative learning approaches. We, thus, decided for the synchronous distance education part, to assess the, reinforced with TBL, effectiveness of the FC approach we adopted. In the following sections our research actions and results are presented.

## Methodology

The educational process that is mainly used by Greek Universities, revolves around teacher-centered instruction, where the presentation of new knowledge takes place in the amphitheater (and/or lab) and study at home. We decided to “flip the amphitheater” because due to the confinement measures, the curricula needed to be transferred online. Therefore, favorable conditions emerged that enable shift from traditional teaching to blended learning environments. For this reason, we implemented the combination of FC and TBL model described below in four Computer Engineering courses. Table 1 illustrates the steps taken to implement the combined FC and TBL approach.

Teachers prepare educational material such as interactive videos and educational resources and make them available to students as sequences of learning activities by using the LAMS LMS (Dalziel, 2003). Videos are considered interactive because students review embedded content, Internet sources and answer questions integrated to them. Teachers record their lectures by selecting an appropriate tool and add interactive content and assessment procedures using Ed-Puzzle and Playposit web-based tools. Students are supposed to study the material at their own pace and complete the assessment procedures included in interactive videos autonomously, before attending on-line classroom.

Online classrooms were materialized via MS-TEAMS synchronous distance education platform. In synchronous sessions, which were held once a week, students posed questions, fulfilled group assignments, and carried out individual and group assessment, always under the guidance of the teacher. To adapt students better to the requirements of the FC, it was sometimes deemed appropriate to dedicate some time of the synchronous session to briefly present demanding concepts of the following section to facilitate individual study. Traditional teaching was minimized in favor of active learning, practice, discussion, self-reflection, and group assignments. We adapted the educational material into sections lasting one to two weeks at most. In both interactive videos and LAMS learning activities we implemented discussion and interaction activities between learners. Teachers encouraged students to engage in discussion and collaboration activities in small groups, formed by the teacher.

At the end of each section, the degree of comprehension of the material is assessed. Assessment is performed by using the interactive videos and LAMS assessment tools. Initially each student individually deals with the assessment procedures and upon completion of the individual assessment the same assessment procedures are performed in groups following a Teams Based Learning (TBL) approach. Τo carry out teams’ assessment, students collaborate in “rooms” during synchronous sessions, moderated by the group leaders and teaching assistants, in order to fulfil the same assessment they previously carried out individually.

Groups are formed by the teacher and lasted during the entire course period. Teachers assess students both individually and in small groups, but they also promote and expect groups to function during the pre-class preparation activities. Group leaders are students who, according to the teacher’s opinion, outperform their colleagues in mastering specific course concepts and can serve as facilitators to help their colleagues in dealing with the educational material. Group assessment is performed by using the Assessment or the Scratchie tool of LAMS. Upon completion of group assessment students have access to direct feedback based on their selections.

Finally, effort was given to ensure the integrity of the educational process by utilizing appropriate LAMS mechanisms (shuffling of multiple-choice questions, appearance of the next task after the completion of that it is depended on, etc.). Upon completion of group assessment, students are asked to evaluate the degree their colleagues in the group contributed to the collective outcome. Teachers consider this intragroup assessment to decide the final grade for each student.

We applied the combined FC/TBL methodology to four Computer Engineering courses. In this section we present the methodology of data collection and processing, as well as the obtained results. The courses are grouped according to their contents and special characteristics:

- Courses DS1 and DS2 concern Distributed Systems and they are both theoretical courses.
- Courses DN1 and DN2 concern Data Networks. The first one is a lab course, while the second is a theoretical course.

The main question investigated, was whether the application of the FC/TBL method in the Emergency Remote Education context improved the learning outcomes for the students.

Theoretical courses used to be taught in the amphitheater and students’ participation was not obligatory, while participation to lab courses was obligatory. For the courses where participation to the lectures was not obligatory, the students were given the option at the beginning of the semester to either participate (a) at the lectures held according to FC/TBL approach or (b) only at the final exams.

The achievements of students that attended courses solely through the combined FC/TBL methodology are compared with the ones of the students that attended classes through face-to-face instruction. It must be stressed that the teaching staff that carried out both afore-mentioned approaches for each course remained the same, while an effort was made for the student assessment activities to be equivalent and comply to the respective learning outcomes for each course. Rstudio 1.4 and MS-Excel were used for data analysis and processing.

## Data collection and results

To investigate the FC and TBL combination effectiveness a comparison of individual Readiness Assurance Test (iRAT) and team Readiness Assurance Test (tRΑΤ) was carried out. An iRAT was answered by the students during the online classroom. Afterwards, a tRAT, identical to iRAT, was answered in groups of students. Various types of questions were contained in both tests (open answer, multiple choice, mapping, etc.) and graded in the range 1–10. To calculate the effectiveness of TBL, we introduce a TBL metric as the percentage of attempts within each course’s activity for which the tRAT score was higher than iRAT score.

$$\text{T}\text{B}\text{L} \text{m}\text{e}\text{t}\text{r}\text{i}\text{c}=\frac{Number of students with tRAT > iRAT}{Total number students}\times 100\%$$

### Distributed systems courses

**Fig. 1:**
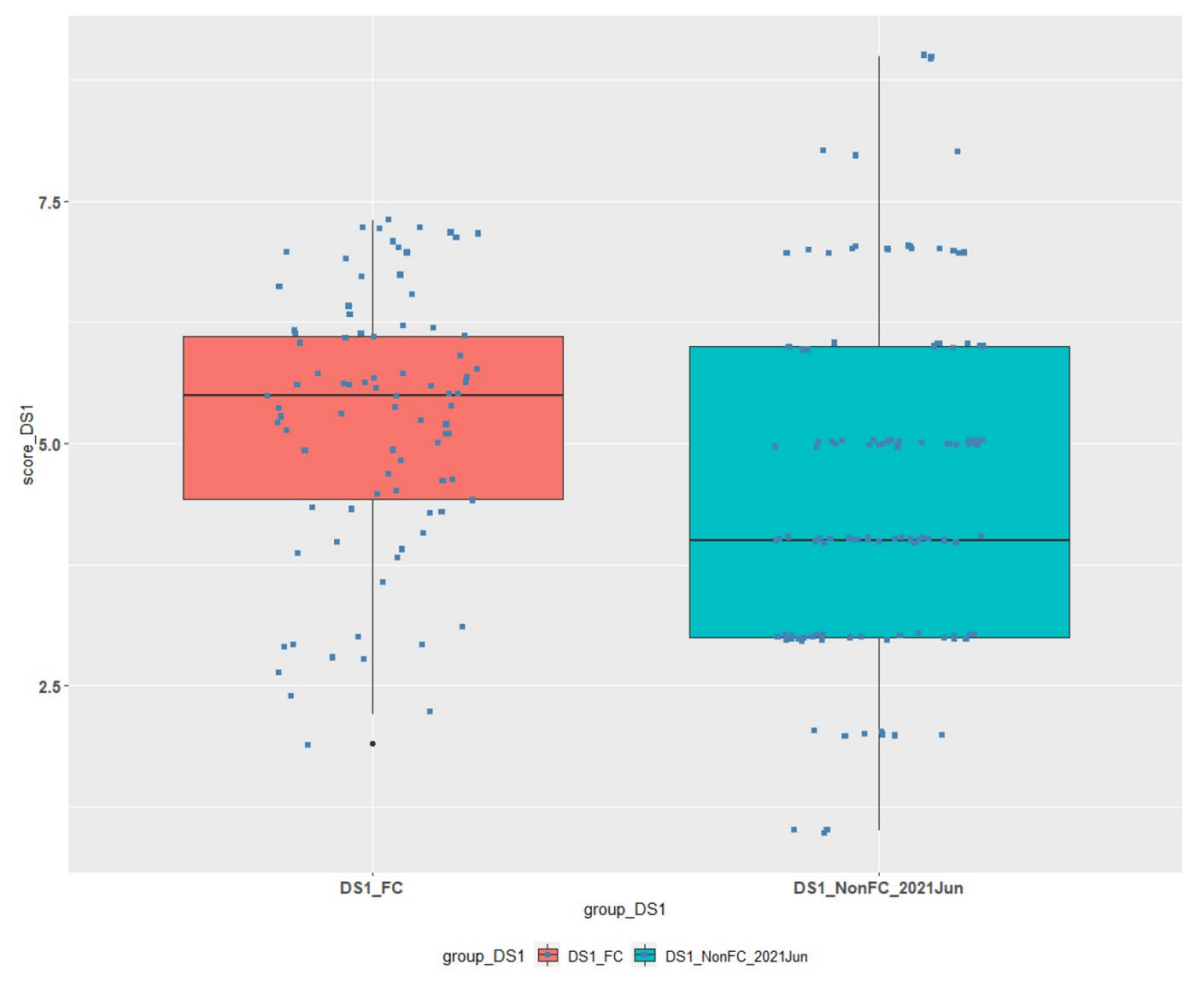
Comparison of FC/TBL and non-FC/TBL students’ achievements for Course DS1.

Table 2 and the boxplots in Fig. 1 illustrate the results for courses DS1 and DS2:

**Table 2:** Descriptive statistics for FC/TBL and non-FC/TBL students’ groups for Courses DS1 and DS2.

Course	Data Group	#N	Min	1st Quantile	Median	Mean	3rd Quantile	Max	SD
DS1	DS1_FC	86	1.90	4.43	5.50	5.23	6.10	7.30	1.35
DS1	DS1_Non_FC	113	1.00	3.00	4.00	4.62	6.00	9.00	1.78
DS2	DS2_FC	27	0.50	5.00	5.60	5.33	6.60	7.60	1.69
DS2	DS2_Non_FC	90	0.50	2.25	3.00	3.15	4.00	6.00	1.29
DS2	DS2_Non_FC2020Feb	77	0.00	1.00	2.0	2.37	3.00	7.70	1.97
DS2	DS2_Non_FC2020Jun	66	0.00	2.50	5.00	4.08	5.50	9.00	2.40
DS2	DS_Non_FC2020Sep	41	0.00	2.50	3.30	3.34	5.00	5.80	1.90

- DS1_FC is our experimental data group for the first course and is compared to DS1_Non_FC data group which contains the grades of students that did not participated in this methodology in June 2021.
- DS2_FC is our experimental data group for the second course and is compared to:
  o DS2_Non_FC data group containing the grades of students from the same semester that **have not** attended the FC/TBL classes.
  o DS2_Non_FC2020Feb, DS2_Non_FC2020Jun, DS_Non_FC2020Sep data groups containing the grades of students from the previous semesters that **have not** attended the FC/TBL process classes.

**Fig. 2:**
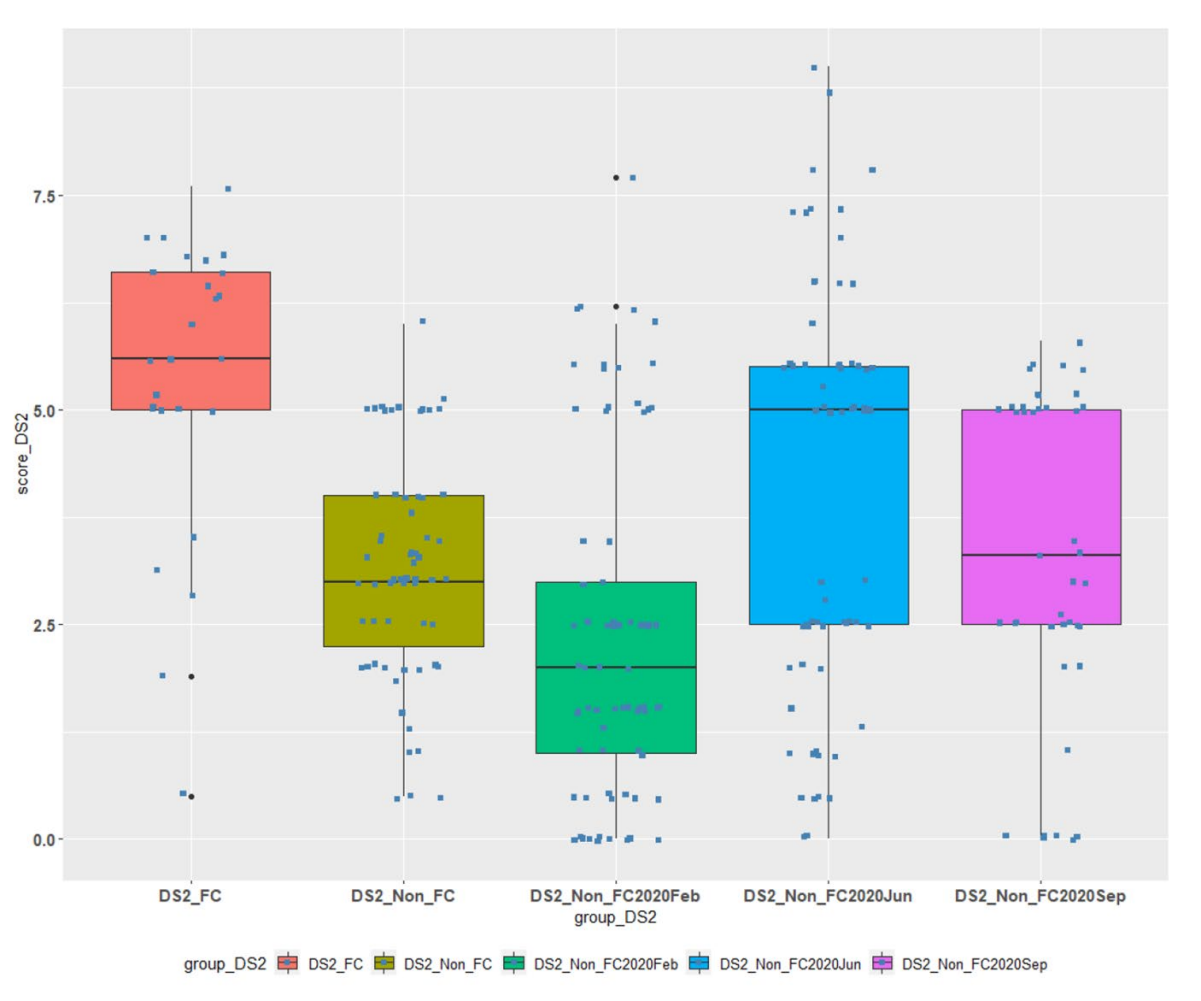
Comparison of FC/TBL and non-FC/TBL students’ achievements for Course DS2.

The verification of statistical difference of the means between the different groups for lesson DS1 was done by Welch Two Sample t-test. It revealed a statistically significant difference of the means at *P = 0.006* for the two groups, thus we observe a considerable improvement in students’ performance when the combined FC/TBL method was applied.

The verification of statistical difference of the means between the different groups for course DS2 was done by Analysis of Variance (ANOVA) and Tukey’s Honest Significant Difference (TukeyHSD) test. These tests revealed a statistical difference at P < 0.05 of the mean for the experimental FC/TBL group (DS2_FC) and all the four comparison groups of examinees mentioned above, i.e., there was an improvement in students’ performance when the combined /TBL method was applied.

### Data networks courses

**Fig. 3:**
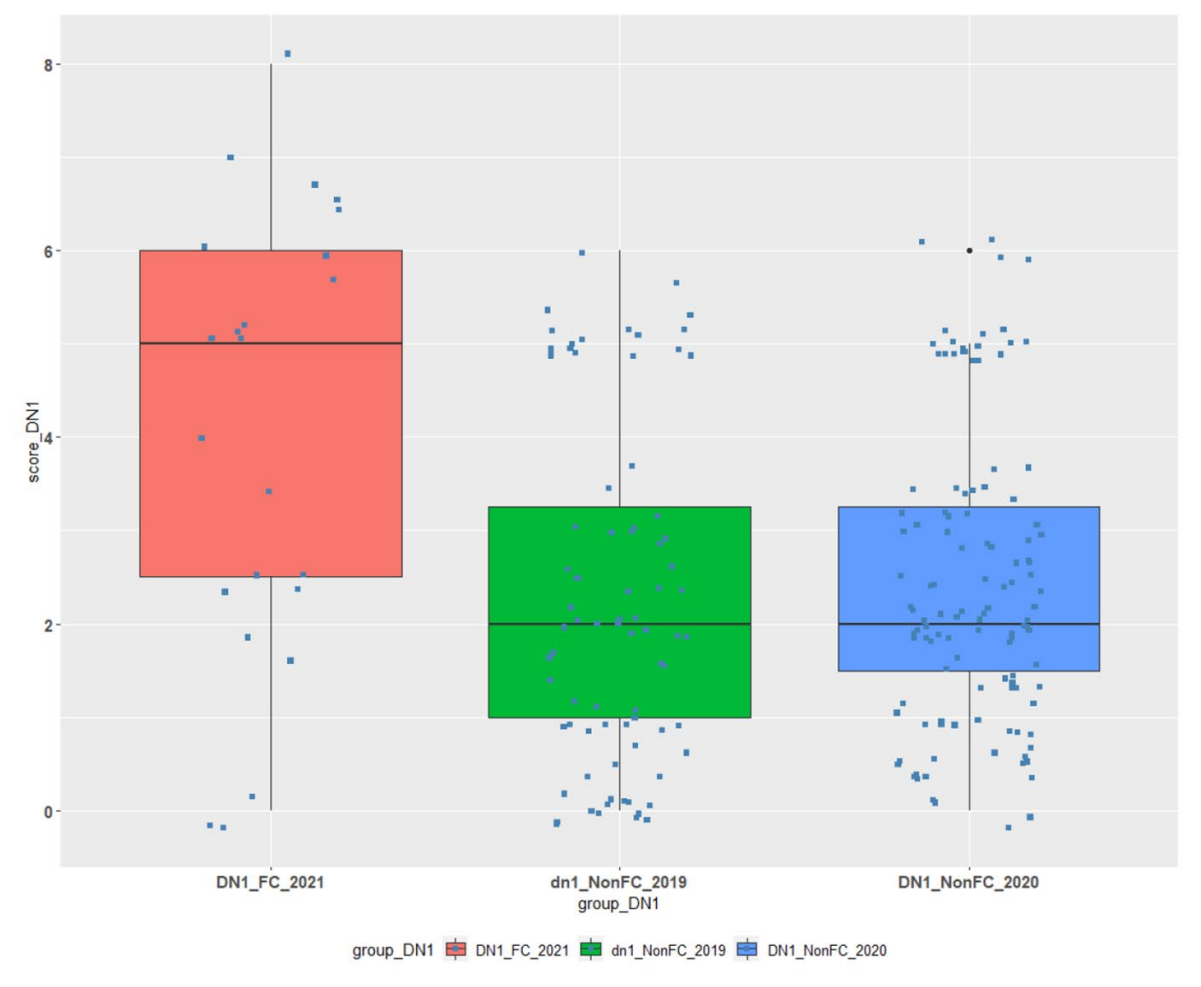
Comparison of FC/TBL and non-FC/TBL students’ achievements for Course DN1.

**Fig. 4:**
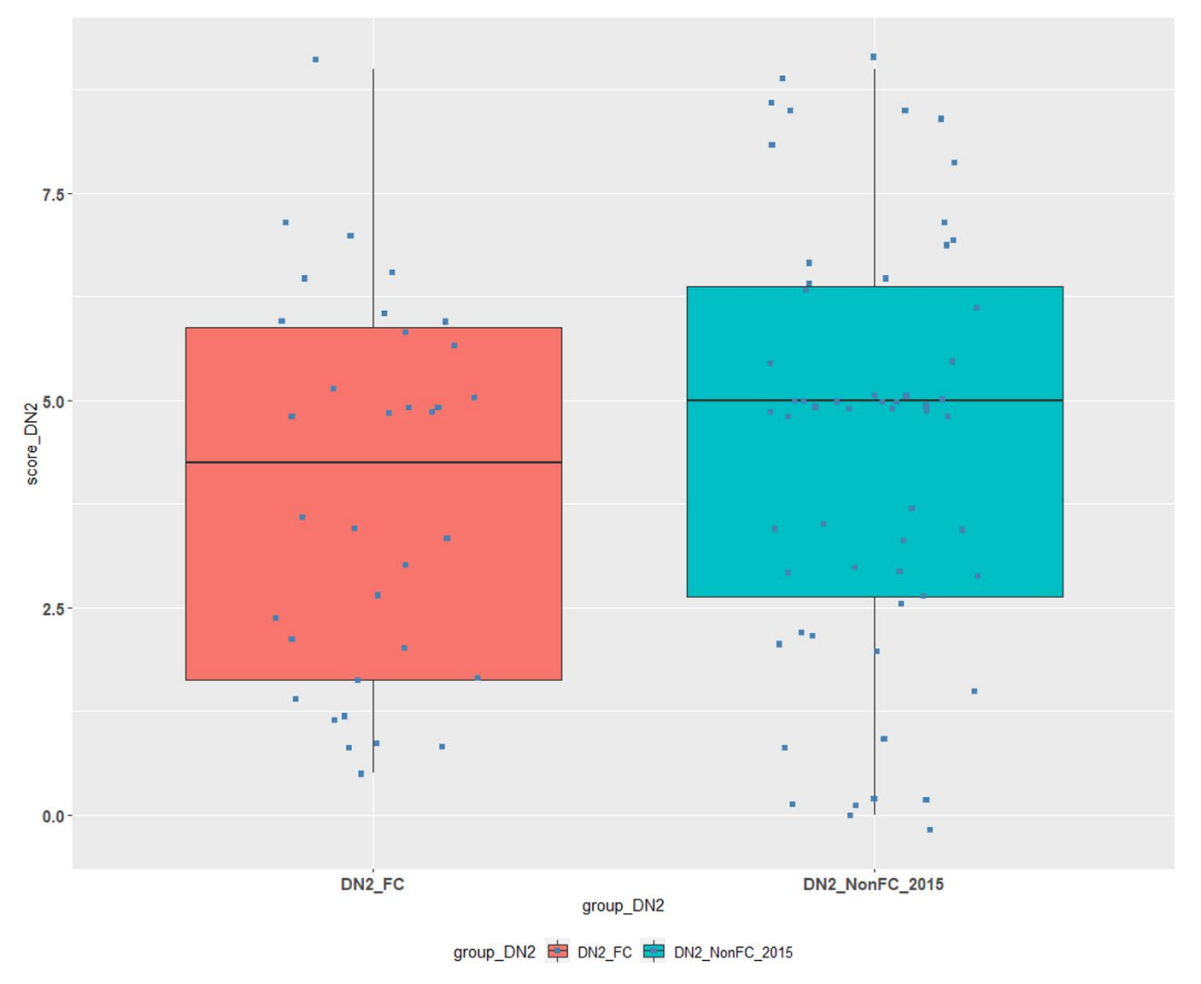
Comparison of FC/TBL and non-FC/TBL students’ achievements for Course DN2.

Similar comparisons are presented below (Table 3; Figs. 3 and 4) for courses DN1 and DN2:

**Table 3:** Descriptive statistics for FC/TBL and non-FC/TBL students’ groups for Courses DN1 and DN2.

Course	Group	#N	Min	1st Quantile	Median	Mean	3rd Quantile	Max	SD
DN1	DN1_FC	23	0.00	2.50	5.00	4.00	6.00	8.00	2.38
DN1	DN1_NonFC_2020	119	0.00	1.50	2.00	2.49	3.25	6.00	1.57
DN1	DN1_NonFC_2019	79	0.00	1.00	2.00	2.29	3.25	6.00	1.83
DN2	DN2_FC	34	0.50	1.63	4.25	3.91	5.88	9.00	2.27
DN2	DN2_NonFC_2015	58	0.00	2.63	5.00	4.45	6.38	9.00	2.56

• DN1_FC, is our experimental data group for the third course containing the grades of students attended the classes with FC/TBL and is compared to DN1_NonFC_2020 & DN1_NonFC_2019 data groups which contain the grades of students that **have not** attended classes with the combined FC/TBL approach.

• DN2_FC, is our experimental data group for the fourth course containing the grades of students attended the classes with FC/TBL and is compared to DN2_NonFC_2015 data group, which contains students’ grades from previous semesters, when the course was taught by the same teaching staff by using traditional face to face methods.

The ANOVA and TukeyHSD test were used to test the means of the DN1 courses’ data groups for statistical differences. The differences in means of DN1_FC_2021 vs. DN1_NonFC_2019 and DN1_FC_2021 vs. DN1_NonFC_2020 were statistically significant (both at *P < 0.001*), indicating that students attended FC/TBL methodology classes had improved performance.

The mean of the experimental data group DN2_FC was lower than the data group DN2_NonFC_2015, however the Welch Two Sample t-test, which was used to test for a significant difference among these groups, revealed that the difference between the mean values is not statistically significant (*P = 0.30*).

### Evaluation of TBL effectiveness

The TBL effectiveness per LAMS activity for the DS1 and DN1 courses are summarized in Table 4.

**Table 4:** TBL metric for courses DS1 and DN1.

Course	Activity	Total TBL Metric	Course	Activity	Total TBL Metric
DS1	1	79%	DN1	1	65%
DS1	2	77%	DN1	2	61%
DS1	3	67%	DN1	3	74%
DS1	4	73%	DN1	4	13%
**DS1**	**Mean**	**74.0%**	**DN1**	**Mean**	**53.2%**

Clearly, for both courses the tRAT results outperform the iRAT results (on average in the 74% of attempts for course DS1 and in the 53% of attempts for course DN1).

### Investigation of students’ perception for the combination of FC and TBL

The students’ perception for the combined approach was investigated by asking the active students of all courses to evaluate certain aspects of the adopted educational process at the end of each course. Table 5 summarizes the main findings of these questionnaires.

**Table 5:** Students’ perception of the combined FC/TBL approach

*Number of students responded, N = 133*				
Question	Totally disagree or disagree	% Totally disagree or disagree	Agree or Mostly Agree	% Agree or Mostly Agree
The workload was increased in classes with combined FC/TBL approach	62	46.6	71	53.4
Online sessions’ content is good or need only minor improvements	17	12.8	116	87.2
The course is now more interesting	17	12.8	116	87.2
Understanding of concepts is high with combined FC/TBL approach	20	15.0	113	85.0
I prefer to learn through similar techniques	23	17.3	110	82.7
I feel very confident for the final exam after attending the FC/TBL class	26	19.5	107	80.5
Video use was very helpful	11	8.3	122	91.7
LAMS technical problems were evaluated as significant obstacles	55	41.4	78	58.6
Your overall experience was positive	15	11.3	118	88.7

It is straightforward from the results of students’ questionnaires that:

- students consider that their understanding was improved, the courses became more attracting, and the use of video was appraised as very helpful. Students reported that the adopted approach increased the required study time however that fact did not prevent them from engaging with the educational process.
- the overall acceptance of the combined FC/TBL approach by the students was very high, considering it as very useful for their studies.

## Discussion

The results presented in the previous sections give evidence that significant difference was observed in students’ achievements for the three out of the four courses when FC is combined with TBL. These results are consistent with most studies that consider application of FC in higher education settings: the trend is that students that attend FC classes are performing better and their engagement in educational activities is improved when compared to students that attend face to face classes.

As it has been pointed out by several studies, there are crucial issues that must be addressed for an online class to be effective. As Van Wart et al., (2020) pointed out, a good online class is one that the instructor provides immediate responses to students and solid learner-to-learner interactions. In our approach TBL Is incorporated in the FC approach, thus interactions are considered of vital importance. We have deployed various tools to foster collaboration between students in the TBL part, such us virtual rooms and forums. Specialized personnel support students in technical aspects of the environment and teachers were always available through e-mail and forums to support students in mastering concepts. As a result, students did not report negative experiences in effective communication between learners and learners and instructors.

A common prerequisite in all the surveys is that the educational environment should encompass methods to engage and challenge students. The combination of online with face-to-face meetings has been proven by many studies a successful motivation (regardless of the Emergency Distance Education situation). iRAT and tRAT tests, that act as a formative assessment method, can also be considered to engage students’ reflection on their progress. Finally, by employing collaborative learning through TBL, students cooperate to answer tRAT tests. Results of tRAT tests are improved when compared to their iRAT counterparts thus revealing upgraded learning outcomes for students and serving as an indication that this upgrade can be attributed to TBL.

Useful conclusions are deduced when our results are compared with similar studies. As similar to ours we consider studies that apply FC approaches in Computer Science or Computer Engineering curricula and study the results by comparing them with the ones of students that attended the same courses in the traditional way. In these works, FC is considered at least equally effective to traditional learning environments. Yang et al. (2019) arrive at the conclusion that a rotational blended learning approach in a Computer Engineering course results in advanced educational outcomes and student satisfaction. Authors applied quasi-experimental methods and descriptive statistics and concluded that the educational outcomes are better for the students that attended FC approach when compared to their control group. Based on the published results, the rotational blended learning outcomes outperform learning outcomes derived by the combined FC/TBL approach.

Troya et al., (2021) demonstrated the merits of an FC approach exclusively in a lab course of a Computer Science curriculum involving 6 instructors and 434 students. They found that FC lends itself to lab courses and motivates students. Even though in our work we deduced that in the DN1 course -an exclusively lab course- the combined FC/TBL approach outperforms the traditional one, Troya et al. reported no statistical evidence on positive influence on students’ grades. As far students’ attitude is concerned, authors concluded, based on descriptive statistics, that students manifest positive attitudes towards lab courses taught with the FC approach.

Similar to the above are the findings of three survey studies (Al-Samarraie et al. (2020); Amresh et al., (2013); Velegol et al., (2015)). Although FC approach seems to place more burden on students’ efforts to cope with the educational material and formative assessment procedures, overall, their performance seems to meliorate and their estimation about the FC approach is positive. These findings are validated by the findings of the combined FC/TBL approach that we presented in the previous sections.

## Conclusions and future work

In this article we present the methodology and results of the application of the Flipped Classroom model combined with Team-based Learning pedagogical strategy in four Computer Engineering courses using Learning Activities Management System (LAMS). The results were encouraging, as there was a positive significant difference in students’ achievements for the three out of the four courses. Moreover, students’ tRAT results outperform the corresponding iRAT results, indicating that the teamwork resulted in improved learning outcomes when compared with the students’ individual achievements. Finally, the perception of the methodology among the students was very high, considering it as valuable for their learning. It is worth noting that the teaching staff observations throughout the courses reinforce the quantitative findings, that the FC/TBL method resulted in better students’ mastering of the material.

Instructors faced some limitations when applying the combined FC/TBL approach. The fact that courses’ education model was rashly shifted to fully distant learning models was an obstacle itself since authors and students had no experience in this way of teaching. Students had also to alter their way of studying and shift from a loose method of assessment (studying mainly a bit before the final exams) to a heavy one (a test at the end of each section, which means a test every two or three weeks). But that worked for their benefit finally, and it is the main reason for their substantial improvement.

We plan to extract information from the collaborative and the discussions tools offered by LAMS, both for intra-communication between the students of a team and between teacher and students. We also plan to further investigate the contribution of each team member to the collective product as a means to assess the outcome of the TBL part of our approach.

Another important issue is to measure the penetration of the team leaders’ opinions in forming answers to the tRAT activities. Considering that team leaders’ role is crucial in the overall team performance and intra-group dynamics, having a knowledge of each team leader’s profile and the degree of his/her influence to the rest of the team can be of importance when selecting team leaders in a way that the overall team performance and communication is maximized.

## Data Availability

Not applicable.

## List of abbreviations

FC: Flipping Classroom
TBL: Team-based Learning
LAMS: Learning Activities Management System
